# Scoping review of complexity theory in health services research

**DOI:** 10.1186/s12913-016-1343-4

**Published:** 2016-03-12

**Authors:** David S. Thompson, Xavier Fazio, Erika Kustra, Linda Patrick, Darren Stanley

**Affiliations:** School of Nursing, Lakehead University, 955 Oliver Road, Thunder Bay, ON P7B 5E1 Canada; Faculty of Education, Brock University, 500 Glenridge Avenue, St. Catharines, ON L2S 3A1 Canada; Teaching and Learning Development, University of Windsor, 401 Sunset Avenue, Windsor, ON N9B 3P4 Canada; Faculty of Nursing, University of Windsor, 401 Sunset Avenue, Windsor, ON N9B 3P4 Canada; Faculty of Education, University of Windsor, 401 Sunset, Avenue, Windsor, ON N9B 3P4 Canada

**Keywords:** Complexity theory, Health services research, Theory

## Abstract

**Background:**

There are calls for better application of theory in health services research. Research exploring knowledge translation and interprofessional collaboration are two examples, and in both areas, complexity theory has been identified as potentially useful. However, how best to conceptualize and operationalize complexity theory in health services research is uncertain. The purpose of this scoping review was to explore how complexity theory has been incorporated in health services research focused on allied health, medicine, and nursing in order to offer guidance for future application. Given the extensiveness of how complexity theory could be conceptualized and ultimately operationalized within health services research, a scoping review of complexity theory in health services research is warranted.

**Methods:**

A scoping review of published research in English was conducted using CINAHL, EMBASE, Medline, Cochrane, and Web of Science databases. We searched terms synonymous with complexity theory.

**Results:**

We included 44 studies in this review: 27 were qualitative, 14 were quantitative, and 3 were mixed methods. Case study was the most common method. Long-term care was the most studied setting. The majority of research was exploratory and focused on relationships between health care workers. Authors most commonly used complexity theory as a conceptual framework for their study. Authors described complexity theory in their research in a variety of ways. The most common attributes of complexity theory used in health services research included relationships, self-organization, and diversity. A common theme across descriptions of complexity theory is that authors incorporate aspects of the theory related to how diverse relationships and communication between individuals in a system can influence change.

**Conclusion:**

Complexity theory is incorporated in many ways across a variety of research designs to explore a multitude of phenomena.. Although complexity theory shows promise in health services research, particularly related to relationships and interactions, conceptual confusion and inconsistent application hinders the operationalization of this potentially important perspective. Generalizability from studies that incorporate complexity theory is, therefore, difficult. Heterogeneous conceptualization and operationalization of complexity theory in health services research suggests there is no universally agreed upon approach of how to use this theory in health services research. Future research should include clear definitions and descriptions of complexity and how it was used in studies. Clear reporting will aid in determining how best to use complexity theory in health services research.

## Background

There are calls to increase the use of theory when designing and conducting health services research. Knowledge translation and interprofessional collaboration are two areas of health services research experiencing such calls. Knowledge translation research is the study of how best to ensure stakeholders are made aware of, and use, research evidence in decision-making [1]. Interprofessional collaboration research explores how best to support professionals to develop and maintain optimal working relationships [2]. Together, knowledge translation and interprofessional collaboration research hold potential for improving health care processes and outcomes [3], nonetheless they share a common criticism. Researchers report low numbers of studies where authors have used theory in their research [4, 5] and such reports have prompted calls for improvement.

Theory is important in designing and conducting both qualitative and quantitative research on phenomena related to health services (e.g., knowledge translation, interprofessional collaboration) as it aids in the development of generalizable and robust knowledge [6, 7]. Explicit use of theory can assist a reader to decide whether findings are applicable and useable in specific settings. Overviews identifying potentially useful theories exist in both knowledge translation and interprofessional collaboration [7, 8]. Authors in both fields suggest that considering theoretical perspectives that include attributes of complexity theory may be useful in a study’s design and data analysis [7, 9–11].

### Complexity theory

Definitions of complexity theory are elusive and “there is no generally accepted statement of what complexity theory is or how complex something must be to come with the ambit of complexity theory” [12]. Conceptual confusion associated with complexity theory may reflect questionable validity, transdisciplinarity [13], and/or lack of in depth knowledge by researchers of the methodological considerations for complexity theory. However, the absence of a universal definition is not akin to an absence of validity. For instance, the transdisciplinary nature of complexity theory is a plausible explanation for an elusive definition because “any definition of complexity is beholden to the perspective brought to bear upon it” [14]. Definitions of complexity are often tailored to reflect the phenomena of interest [15]. Despite authors using complexity theory, little is known on how to conceptualize and operationalize this theory to best suit health services research. For the purpose of this review, we align ourselves with Cilliers’ [16] description of complexity theory: “complexity is a characteristic of a system”. Specifically, for this review, we view complexity theory as a perspective that conceptualizes relationships of components (i.e., individuals) within a system as the foundation from which the properties of a system emerge.

Drawing from Cilliers [16], and Strumberg and Martin’s [17] work, we offer some propositions of complexity theory. First, complexity theory offers a perspective to studying complex systems in a manner that does not reduce the system to individual components. From a complexity theory perspective, the interactions between components of a system are important for studying a system. Second, it is the interactions of system components that result in the overall behavior of the system. Complexity theory acknowledges that agents within a system interact to produce such behavior. Using complexity language, self-organization refers to the interactions between agents and emergence refers to the system level changes. Third, the interactions between agents are not controlled by a central control. Interactions arise from individual agents following simple rules and responding to environmental changes—control is decentralized. Fourth, the system is open to the surroundings. Interaction of the agents with their surroundings results in the exchange of information and people. These exchanges influence how those agents interact. Finally, agents have limited control over how system level changes emerge. As such, new system behavior is often unpredictable and difficult to trace back to a specific cause. These propositions, while not exhaustive, offer a general understanding of complexity theory for the purposes of our review.

Reviews of complexity theory exist in organizational science [18], mathematics and management [19], and health care [20]. Wallis [18] examined how complexity was used in the organizational science literature and concluded there was great diversity in application. In turn, he called for a more explicit and comprehensive application of the concepts of complexity. Pollack et al. [19] compared the use of complexity theory between mathematics and organizational science research. They found researchers in organizational science, although late adopters of complexity theory when compared to researchers in mathematics, are continuing to explore ways of applying complexity theory to management questions. These findings were consistent with a review by Sturmberg et al. [20] exploring the evolution of family medicine/general practice from a complex systems perspective. Like Pollack et al [19], Sturmberg et al. [20] found researchers were applying complexity theory more frequently than several decades ago. Notwithstanding, social science researchers use complexity in a metaphorical manner whereas computer science and mathematics use complexity for quantitative modeling. Across all three reviews, conclusions suggested that the “proper” or “feasible” application of complexity to social contexts remains unknown.

Researchers are increasingly incorporating complexity theory in health services research despite ongoing debate on how best to do it [21–23]. There are no reviews exploring how complexity theory has been incorporated in the broader health services research literature related to nursing, medicine, and allied health. Given the extensiveness of how complexity theory could be conceptualized and ultimately operationalized within health services research, a scoping review of complexity theory in health services research is warranted.

The purpose of this scoping review is to explore how complexity theory has been incorporated in health services research. In doing so, we answer the following research questions:

1. What are the characteristics of studies that use complexity theory in health services research?
2. What settings and professions do researchers study using complexity theory?
3. What research questions and phenomena of interest do researchers focus on when using complexity theory?
4. How are researchers using complexity theory within health services research^1^?
5. How are researchers describing complexity theory within health services research?

## Methods

We anticipated heterogeneous studies in terms of research purposes, phenomena of interest, methods, participants, and context. Likewise, although we aimed to conduct a broad, replicable, and systematic search of published literature, we did not seek to appraise and synthesize research evidence. Therefore, a systematic review was not warranted. In an evaluation of review methods, Grant and Booth [24] described scoping reviews as “a preliminary assessment of potential size and scope of available research literature”. Arksey and O’Malley [25] and Levac, Colquhoun, and O’Brien [26] have developed and advanced the recommended methodological framework for scoping reviews [27]. Scoping reviews involve five steps: (a) identifying the initial research question; (b) identifying the relevant studies; (c) selecting the studies; (d) charting the results; (e) collating, summarizing, and reporting the findings; and (f) consulting stakeholders for knowledge translation of findings [25]. With the exception of consultation of stakeholders, we followed Arksey and O’Malley’s approach, and used Levac et al. as a guide, for how to operationalize each step.

### Identifying relevant studies

Literature published between inception of each database and June 2015 was collected from the following databases: The Cochrane Database of Systematic Reviews, CINAHL, EMBASE, Medline, and Web of Science. The search strategy and database selection was determined in consultation with a Master of Library Information Science (MLIS) Librarian and a researcher familiar with complexity theory (DS). Table 1 outlines the search strategy for each database. Given the breadth of complexity theory, combined with a lack of agreed upon nomenclature, we anticipated literature to be indexed under a variety of terms. To account for broad indexing, we used a range of search terms often associated with complexity theory. We used citation searching when key articles were found.

**Table 1:** Search strategy by database

Database	Search strategy
CINAHL	Complexity theory OR complexity science OR complex adaptive system OR complexity thinking OR complex responsive process theory OR chaos theory
Cochrane Database of Systematic Reviews	Complexity theory OR complexity science OR complex adaptive system OR complexity thinking OR chaos theory OR complex responsive process theory
EMBASE	Complexity theory OR complexity science OR complex adaptive system OR complexity thinking OR chaos theory OR complex responsive process theory
Medline	Complexity theory OR complexity science OR complex adaptive system OR complexity thinking OR chaos theory OR complex responsive process theory
Web of science	TS = (“complexity theory” OR “complexity science” OR “complex adaptive system” OR “complexity thinking” OR “complex responsive process theory” OR “chaos theory”) *DocType = All document types; Language = All languages*

### Study selection

A study was eligible for inclusion if: (a) it was published in a peer-reviewed journal, (b) it was written in English, (c) authors provided a statement somewhere in their manuscript reporting they incorporated complexity theory within their research, (d) authors studied a phenomena related to health services research, and (e) authors included nurses, physicians, or allied health professionals.

For *criterion c* we did not exclude studies on the basis of study design.

Articles describing quality improvement projects were excluded, but articles describing quality improvement research or research on quality improvement techniques were included. We excluded articles describing quality improvement projects because the focus of quality improvement projects differs from that of research, with the former focused on descriptions of how a group worked to improve care for a specific population or organization and the later focused on developing new and (often) generalizable knowledge [28]. Our focus is on complexity theory in health services *research*, thus we excluded descriptions of quality improvement projects. Distinguishing between quality improvement and research reports is difficult [29]. To assist, we used criteria described by Newhouse et al. [28] that included assessment of intent of the authors, burdens and risks to subjects, and oversight of the project.

For *criterion d*, we used the Canadian Institute of Health Research definition of health services research [30]. We excluded studies that used complexity theory to explain aspects of diseases (e.g., atrial fibrillation, cerebral vascular accidents). Likewise, we excluded studies offering commentary or discussion articles on how complexity theory could be used in research.

For *criterion e* we defined allied health professionals as dietitians, occupational therapists, pharmacists, physiotherapists, and speech-language pathologists. If studies involved more than the seven professions listed above, they were included only if they focused primarily on nurses, physicians, or allied health professionals. For studies with multiple professions, when possible, we included only the results pertaining to the seven professions above. Studies were excluded if they focused solely on pre-licensure students. We had no historical date limits.

DT independently screened titles and abstracts. Articles that met inclusion criteria were then reviewed a second time using full text. If questions arose related to article eligibility, a second author (DS) reviewed the article. The second author (DS), who is familiar with the complexity literature, reviewed the final list of included studies. The list of articles was sent to a third party expert in the field of complexity for review. All studies were imported into and managed with bibliographic software (Zotero™).

### Charting the data

Consistent with Arksey and O’Malley [25], we extracted data related to answering our research questions. Data was entered into a Microsoft Excel spreadsheet and individual tables constructed for analysis. Data included authorship, publication year, country of research, research design, professions involved, setting of research (e.g., long term care, acute care), interprofessional focus, purpose/objective of research, attributes of complexity theory used, phenomena of interest, how complexity theory was used, and definition/description of complexity theory provided. In keeping with a scoping review approach, we did not assess the methodological quality of included studies.

### Collating results

According to Arksey and O’Malley [25], a framework should be used to collate results. We created a framework guided by our five research questions. First, we created a data table for study characteristics, including first author, year published, country, and study design. Second, we created a data table outlining the professions involved, the area of research, the setting of research, and whether the research focused on interprofessional collaboration or education. From these tables we compared characteristics, setting, and profession across all studies to answer our first two research questions. Third, we categorized studies based on their research purpose using the verb presented by the researcher(s) in their purpose statement (e.g., describe, explain, explore). While verbs may overlap when referring to research purposes (e.g., describe and explore), we categorized based on how the authors described their purpose regardless of potential overlap to minimize subjective interpretation of purpose. We then determined each researcher’s phenomena of interest. Specifically, we reviewed all research purposes and identified common phenomena of interest. This provided us with a means to categorize studies by research purpose and then compare how the phenomena of interest differed within and between each category thus answering our third research question. Fourth, we reviewed each study and identified how researchers used complexity theory in their study (e.g., conceptual framework, data analysis, interpret findings). Collectively, this approach allowed us to answer our fourth research question. Finally, we created a data table containing the description of complexity theory from each study. From this, we determined the attributes of complexity theory used by each group of authors. To organize the attributes, we followed an approach used by Wallis [13] in his review of complexity in the organizational theory literature. Specifically, we extracted descriptions of the attributes (i.e., conceptual components) of complexity from the definitions and descriptions provided by the authors of the studies in our review and grouped attributes together when authors were describing the same thing. For example, we combined *relationships* and *connections* as one attribute: *relationships*.. We then looked for common themes between descriptions.

## Results

**Fig. 1:**
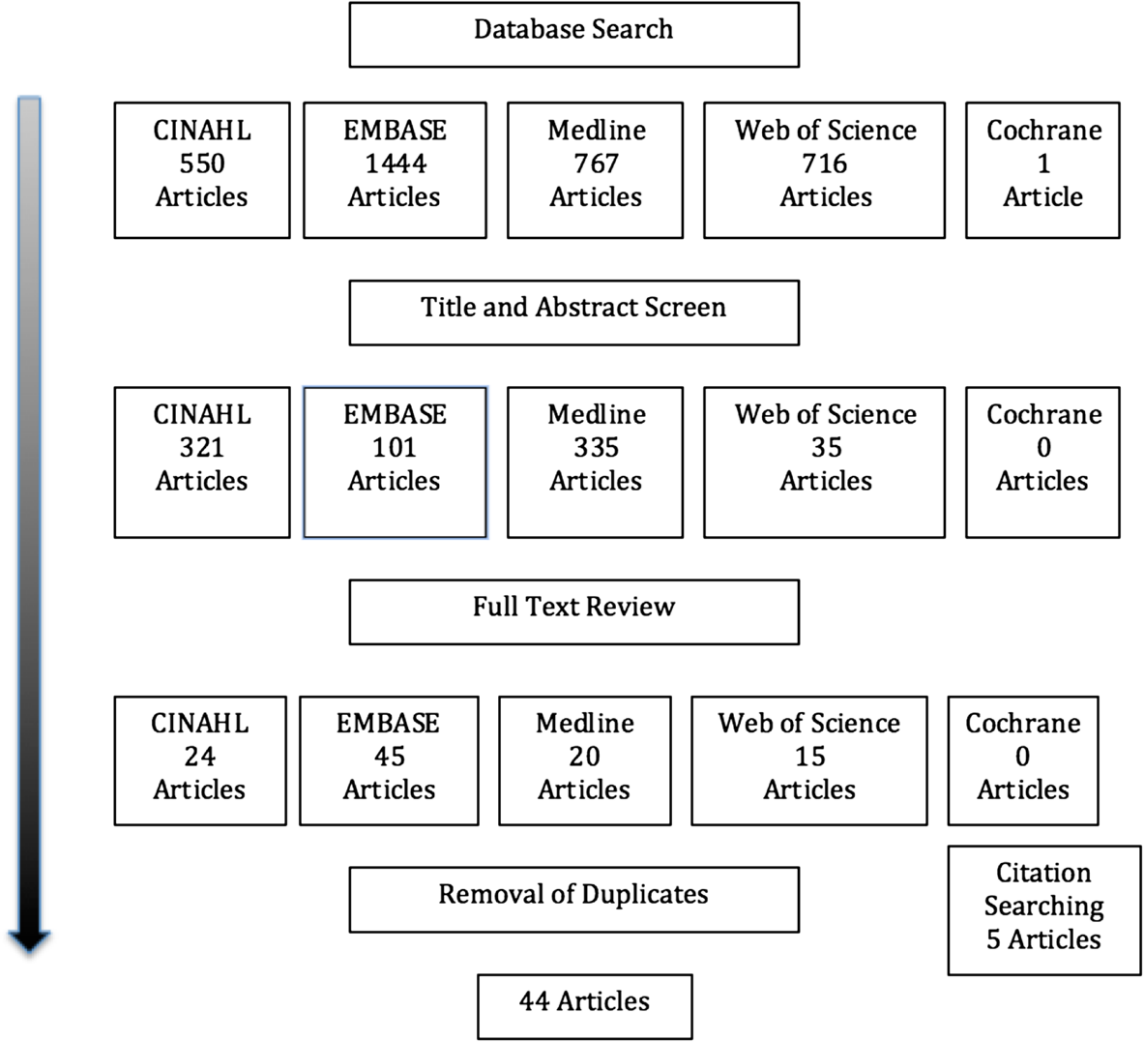
Search and retrieval results

Figure 1 provides an overview of the search and retrieval results. 3478 citations were found by our search strategy. After reviewing titles and abstracts, 792 articles remained. Full text review resulted in 104 articles and after removal of duplicates (*n =* 55) and citations searching (*n =* 5), 44 articles were included in our review. Common reasons for study exclusion included: (a) the article was a commentary or debate on the use of complexity theory, (b) the authors used complexity theory to describe an aspect of a disease (e.g., the neural pathway changes of Parkinson’s Disease), (c) the study included participants not in our inclusion criteria (e.g., pre-licensure learners, administrators) or (d) the research focus not related to health services research (e.g., acoustic properties in rabbits within the context of hearing and speech research).

### Characteristics of studies using complexity theory

The general characteristics of studies incorporating complexity theory in health services research are outlined in Table 2. Most studies were qualitative [31–56], followed by quantitative [57–70], and, finally, to a lesser extent, mixed methods [71–73]. Case studies were the most common qualitative [31, 32, 34–37, 40–42, 44, 45, 47, 50, 51, 55, 63, 74] and mixed method [71–73] design. Action research [48], ethnography [39], grounded theory [46, 53], and phenomenological designs [43, 49] were used less frequently. Two authors did not identify a specific qualitative design [54, 56]. There was a mix of designs across the quantitative studies including, in order of frequency, cross-sectional [57, 58, 60, 66, 69], randomized controlled trials [61, 62, 70], retrospective [64, 67], prospective cohort [63], systematic review [65], and unclear [68]

**Table 2:** Study characteristics, application, and attributes of complexity theory in health services research

First author	Year	Country	Setting	Professions involved	Research design	Use of complexity theory	Attributes of complexity theory used
Aita [31]	2005	USA	Primary Care	Physicians	Qualitative—Secondary Analysis of a Comparative Case Study	Data analysis	Attractors
Anderson [57]	1999	USA	Long Term Care	Nurses	Quantitative—Cross Sectional	Conceptual framework and interpretation of findings	Communication, Connections, Diversity
Anderson [58]	2003	USA	Hospital	Nurses, Pharmacists, and Physicians	Quantitative—Cross Sectional	Conceptual framework	Connections, Diversity, Feedback
Anderson [59]	2003	USA	Long Term Care	Nurses	Quantitative—Cross Sectional	Conceptual framework	Communication, Connections, Diversity, Self-Organization
Anderson [60]	2004	USA	Long Term Care	Nurses	Quantitative—Cross Sectional	Conceptual framwork	Connections, Communication, Self Organization
Anderson [32]	2014	USA	Long Term Care	Nurses	Qualitative—Comparative Case Study	Conceptual framework and interpret findings	Communication, Connections, Diversity, Emergence, Non-Linearity, Self-Organization
Brandstorp [33]	2015	Norway	Primary Care	Nurses and Physicians	Qualitative—Action Research	Data analysis	Attractors, Adaptation, Emergence, Feedback, Self-Organization
Brannon [34]	2009	USA	Long Term Care	Nurses	Qualitative—Case Study	Data analysis	Agents, Connections, Diversity, Emergence, Feedback, Self-Organization
Buttigieg [35]	2013	Malta	Rehab Hospital	Physiotherapists, Occupational Therapists, Nurses, Pharmacists,	Qualitative—Case Study	Conceptual framework	Unclear
Colón-Emeric [61]	2013	USA	Long Term Care	Nurses	Quantitative—Cluster Randomized Control Trial	Conceptual framework	Communication, Connections, Diversity, Self-Organization
Colón-Emeric [36]	2006	USA	Long Term Care	Dieticians, Nurses, Physiotherapists, Occupational Therapists	Qualitative—Case Study	Data analysis	Adaptation, Communication, Diversity, Self-Organization
Cólon-Emeric [37]	2006	USA	Long Term Care	Nurses and Physicians	Qualitative—Comparative Case Study	Conceptual framework	Connections, Communication, Diversity, Learning
Cucolo [38]	2015	Brazil	Hospital	Nurses	Qualitative—Content Analysis	Data analysis	Unclear
Dickinson [62]	2014	USA	Community Health Centres and Primary Care	Physicians	Quantitative—Cluster Randomized Control Trial	Conceptual framework	Adaptation, Connections, Diversity, Learning, Reflection
Eika [39]	2015	Norway	Long Term Care	Nurses	Qualitative—Ethnography	Conceptual framework	Emergence, Learning, Self-Organization
Ellis [40]	2010	United Kingdom	Primary Care	Nurse and Physicians	Qualitative—Comparative Case Study	Interpret findings	Adaptation, Agents, Co-Evolution, Self-Organization
Ellis [71]	2011	United Kingdom	Primary Care	Nursing and Physicians	Mixed Methods—Case Study	Interpret findings	Agents, Co-Evolution, Emergence, Self-Organization
Ellis [41]	2011	United Kingdom	Primary Care	Nursing and Physicians	Qualitative—Case Study	Interpret findings	Adaptation, Agents, Co-Evolution, Self-Organization
Erdek [63]	2004	USA	Hospital	Nurses and Physicians	Quantitative—Prospective Cohort Study	Interpret findings	Unpredictability
Essen [72]	2013	Sweden	Rheumatology Registry	Physicians and Nurses	Mixed Methods—Case Study	Data analysis and interpret findings	Equilibrium, Emergence, Feedback, Self-Organization
Forbes-Thompson [42]	2007	USA	Long Term Care	Nurses	Qualitative—Case Study	Data analysis	Communication, Connections, Diversity
Ford [73]	2009	USA	Hospital	Nurses	Mixed Methods—Case Study	Interpret findings	Diversity, Emergence, Relationships
Glenn [43]	2014	USA	Hospital	Nurses	Qualitative—Hermeneutic Phenomenology	Conceptual framework and interpret findings	Agents, Decentralized Control, Emergence, Feedback, Non-Linearity, Self-Organization
Haigh [64]	2008	United Kingdom	Hospital	Nurses	Quantitative—Retrospective Statistical Modeling	Equation to predict changes	Attractors, Equilibrium, Non-Linearity
Hilts [44]	2013	Canada	Primary Care	Physicians	Qualitative—Case Study	Data analysis	Communication, Emergence, Reflection
Karemere [45]	2015	Congo	Hospitals	Physicians	Qualitative—Case Study	Data analysis	Agents, Path Depenedency, Transition Phase
Lanham [46]	2009	USA	Primary Care	Physicians	Qualitative—Secondary Analysis Grounded Theory	Data analysis	Agents, Connections, Diversity, Emergence, Learning
Lanham [74]	2013	USA	Hospitals and Community	Nurses	Qualitative—Case Study	Interpret findings	Connections, Learning, Self-Organization
Leykum [65]	2007	USA	Not Applicable	Studies that would include at minimum nurses and physicians	Quantitative—Systematic Review	Classification	Co-Evolution, Connections, Learning, Self-Organization
Longo [47]	2007	Italy	Primary Care	Physicians	Qualitative—Case Study	Conceptual framework and interpret findings	Learning, Relationships
Mash [48]	2008	South Africa	Community Health	Nurses and Physicians	Qualitative—Action Research	Interpret findings	Emergence, Self-Organization, Relationships
Matthews [49]	2007	United Kingdom	Health Trusts	Nurses, Physicians, Pharmacists	Qualitative—Phenomenology	Conceptual framework and intertpret findings	Agents, Diversity, Emergence, Feedback, Non-Linearity, Self-Organization
Miller [50]	2001	USA	Primary Care	Nurses and Physicians	Qualitative—Comparative Case Study	Data analysis	Co-Evolution, Emergence, Self-Organization
Oyeleye [66]	2013	USA	Hospital	Nurses	Quantitative—Cross-Sectional	Conceptual framework	Agents, Non-Linearity, Relationships
Pitkäaho [67]	2015	Finland	Hospital	Nurses	Quantitative—Retrospective	Conceptual framework	Feedback, Non-Linearity, Relationships
Piven [51]	2006	USA	Long Term Care	Nurses	Qualitative—Case Study	Data analaysis	Communication, Connections, Diversity
Provost [52]	2015	USA	Hospitals	Nurses, Pharmacists, Physicians	Qualitative—Field Study	Conceptual framework	Communication, Learning, Relationships
Rangachari [53]	2008	USA	Hospital	Physicians	Qualitative—Grounded Theory	Conceptual framework	Attractors, Diversity, Emergence
Rantz [54]	2013	USA	Long Term Care	Nurses	Qualitative—Unclear	Conceptual framework and data analysis	Connnections, Communication, Emergence, Self-Organization
Rantz [70]	2012	USA	Long Term Care	Nurses	Quantitative—Randomized Controlled Trial	Conceptual framwork	Communication, Connections, Diversity
Ruhe [55]	2005	USA	Primary Care	Physicians	Qualitative—Case Study	Data analysis	Communication, Connections, Diversity, Emergence, Equilibrium, Feedback
Singh [68]	2004	USA	Primary Care	Nurses and Physicians	Quantitative—Unclear	Conceptual framework	Adaptation, Central Attractors, Communication, Diversity
Sterns [69]	2010	USA	Long Term Care	Nurses	Quantitative—Cross Sectional	Classification	Agents, Unpredictability
Tsasis [56]	2012	CAN	Health Care System	Nurses and Physicians	Qualitative—Unclear	Data analysis	Agents, Co-Evolution, Diversity, Emergence, Non-Linearity, Self-Organization

Note: Only the professions outlined in our elegibility criteria are reported in Table 2

.

The majority of health services research conducted using complexity theory was based in the United States [31, 32, 34, 36, 37, 42, 43, 46, 50–63, 65, 66, 68–70, 73, 74], followed by the United Kingdom [40, 41, 49, 64, 71], Canada [44, 56], Norway [33, 39], Brazil [38], Congo [45], Finland [67], Italy [47], Malta [35], South Africa [48], and Sweden [72].

### Settings and professions studied using complexity theory

All of the seven professions listed in our inclusion criteria were represented in our review. Authors in 70 % of the studies included more than the seven professions that comprised our inclusion criteria, with management being the most common group in addition to our inclusion criteria. Studies including nursing were most frequent (82 %) followed by studies including physicians (52 %).

The settings studied using complexity theory consisted of long term care facilities [32, 34, 36, 37, 39, 42, 51, 54, 57, 59–61, 69, 70], primary care [31, 33, 40, 41, 44, 46, 47, 50, 55, 62, 68, 71], hospital [35, 38, 43, 45, 52, 53, 58, 63, 64, 66, 67, 73, 74], community health centres [48, 62, 74], and other (e.g., not applicable, health care systems, health trusts) [49, 56, 65, 72]. Despite most of the research being conducted with multiple professions and in settings that depend upon interprofessional collaboration, only 23 % of studies used complexity theory to explicitly explore interprofessional collaboration.

### Research purpose and phenomena of interest

Authors used a variety of research purposes to study an assortment of phenomena using complexity theory. See Table 3 for research purposes and phenomena grouping for all studies. The most common research purpose was exploratory (30 %). Of these, 69 % of studies listed a second purpose (to test, to describe, to develop, to examine, to identify). We further grouped exploratory studies into categories based on their phenomena of study (Table 3). These included interactions/relationships (e.g., participation in decision making [57]), management (e.g., management practices on staff turnover [60]), working environment (e.g., staff perspectives on caring practices [43]), and leadership (e.g., training teams [33]). Two studies had two phenomena of interest based on our coding scheme [44, 58]. Authors of one study [58] explicitly focused on both management and interactions/relationships and the other study [44] explicitly focused on working conditions and change. All of the exploratory studies involving interactions/relationships focused on health professionals.

**Table 3:** Research purpose and phenomena of interest

Research purpose	Phenomena of interest
Exploratory	Change [41, 44], Leadership [33], Management [48, 58, 60], Interactions/Relationships [39, 49, 52, 53, 57, 59], Working environment [43, 44, 66]
Describe	Interactions/Relationships [32, 36, 37, 51, 54], Management [32, 36, 40], Working environment [42]
Examine	Change [65, 69, 74], Interactions/Relationships [31]
Combined other purposes	Change [34, 47, 55, 56, 62, 68, 70–72], Management [35, 41, 64, 73], Interactions/Relationships [46, 61, 67], Working Environment [38, 45, 50, 56]

Research purposes aimed at describing phenomena were the second most common (16 %). Of these, two studies [36, 51] listed a second purpose of exploring. Similar to the exploratory studies, we grouped studies based on the phenomena of interest. Similar to the exploratory studies, the majority of descriptive studies aimed to describe an aspect of interaction/relationships (e.g., describe staff behaviour in group processes [54]) between health professionals either as a primary aim or as a combined aim with management (e.g., describe connection patterns among staff [36]). One study described aspects solely related to management (e.g., clinical governance, management practices [40]) and one study described aspects solely related to work environment (e.g., describe working conditions in nursing homes [42]).

Research purposes aimed at examining phenomena were the third most common (9 %). Due to the low number of studies, we narratively report the results. The first group of authors [31] examined interactions/relationships. Specifically, they examined features of practice related to patient centeredness using a secondary analysis of qualitative data. They concluded that attributes of complexity theory assisted them in examining how patient centeredness occurs within patient and physician interactions. The second group of authors [65] examined change. They conducted a systematic review of interventions aimed at improving Type II diabetes. The authors assigned a value to each intervention based on the degree of complexity that the intervention exhibited. The authors used the degree of complexity to examine whether interventions based on complexity attributes were more effective than interventions that were not based on complexity. They concluded that interventions with a greater number of complexity attributes were more effective for changing diabetic outcomes. The third group of authors [69] also examined change. These authors examined the degree of culture change practice adoption. They ranked culture change practices based on their degree of complexity and examined the degree of adoption. The authors concluded that less complex practices may be easier to implement and that implementation of less complex practices may improve implementation of more complex changes. Finally, Lanham and colleagues [74] used several attributes of complexity theory to re-examine two studies that evaluated the spread of effective interventions. They concluded that self-organization, sense making, and interconnections could be used to facilitate the spread of effective practices.

The heterogeneity of research purposes included in the remaining studies (45 %) prevented meaningful comparison. The research purposes that authors reported include advance and understand, analyze, compare, demonstrate, determine, document, estimate impact, evaluate, identify, implement, improve, produce, suggest, test hypothesis, and understand. We categorized these studies based on phenomena of interest. Change was the most common focus of studies within this category, followed by work environment, management, and, finally, interactions/relationships.

In summary, based on our analysis of research purpose and phenomena of interest, studies aimed at *exploring* and studies aimed at *describing* represent the most common research purpose of health services research incorporating complexity theory. Within these categories, complexity theory was incorporated primarily to explore or describe interactions/relationships between health care workers. There is a wide range of research purposes in the remaining studies. Within these remaining studies, the most common phenomenon of interest was change.

### Use of complexity theory in health services research

Researchers have used complexity theory in their research in a variety of ways (Table 2). The most common was as a conceptual framework applied to research approach and design (45 %).^2^ Examples include using complexity theory to conceptualize variables that were subsequently operationalized to determine if attributes of complexity account for rates in staff turnover [60], using complexity theory to conceptualize the work environment [43], and using complexity theory to conceptualize primary care organizations [40]. There was variation on how explicit authors were regarding how they used complexity theory as a conceptual framework. Some authors described in detail the attributes they used and how they used them, whereas others stated that their research incorporated a complexity framework without describing which attributes or how complexity was used (e.g., [45]).

The second most common use of complexity theory was as a framework for data analysis (32 %). In this group, all studies were qualitative designs and the majority (57 %) were case studies with authors using attributes of complexity to in data analysis. Examples of how complexity theory were used to in data analysis include comparing attributes of complexity (e.g., self organization, emergence) across case studies [50], using complexity to “understand what we were seeing” [31], and using complexity to code observations [34]. Again, similar to those that used complexity as a conceptual framework, authors who used complexity as a data analysis framework varied in detail regarding what they used and how they used it.

Finally, the third most common use of complexity theory was as a framework for interpreting findings (29 %). Examples include using complexity to illustrate leadership principles [73], explain clinical governance [40], and hypothesize why an intervention worked to improve pain control [63].

The remaining three studies used complexity to predict change [64] or classify either interventions [65] or culture change practices [69]. Several authors reported dual applications of complexity (e.g., [47]) and we included both applications in our results (Table 2).

### Descriptions of complexity theory

Authors have incorporated a wide range of attributes from complexity theory to study phenomena related to health services research. To facilitate analysis, we grouped certain attributes into categories when authors appeared to refer to the same (or similar) concept of complexity. Table 4 lists the referent attributes we combined and the term we used to refer to the parent attribute. Wallis [18] used a similar approach in his review of complexity theory in organizational science. As complexity theory has no agreed upon definition and a myriad of concepts that comprise the theories subsumed within complexity theory, it was necessary to combine certain attributes to facilitate analysis. Furthermore, it is beyond the scope of this review to offer a definition of each attribute. However, readers interested in definitions/descriptions of attributes of complexity may be interested in referring to The Handbook of Systems and Complexity in Health [75].

**Table 4:** Parent and referrent attributes

Parent attribute	Referent attributes
Connections	Connections, Relationships, Interconnections
Communication	Communication, Conversation, Information Flow, Information Exchange, Interactions
Learning	Learning, Sense Making, Learning Culture
Adaptation	Adaptation, System Adaptation, Innovation
Diversity	Diversity, Cognitive Diversity, Diversity of Information, Diversity of Perspective, Diversity of Views
Equilibrium	Equilibrium, Disequilibrium
Agents	Agents, Agents in a System, Input from Agents
Unpredictability	Unpredictability, Uncertainty, Levels of Certainty

Overall, researchers incorporated a total of 18 attributes when referring to complexity theory (Table 2). All of the studies except for two [31, 63] incorporated a combination of attributes. Aita and colleagues [31] incorporated the concept of attractors to interpret secondary data and explore what is involved in patient-centered care within primary care settings. Erdeck and Pronovost [63] introduced an intervention aimed at improving pain management that incorporated the concept of unpredictability (i.e., varying levels of certainty). Notably, in two studies, it was unclear what attributes of complexity the authors used [35, 38].

A combination of three or four attributes of complexity theory was most common. The most attributes incorporated by a group of authors was six. This was done by six groups of authors [32, 34, 43, 49, 55, 56]. Within this group, emergence was included in all studies, followed by self-organization, feedback, agents within a system, non-linearity, and diversity. The remaining attributes appeared once or twice in various combinations.

For all studies included in this review, the most common attributes of complexity theory were relationships (*n =* 21), self organization (*n =* 19), diversity (*n =* 19), emergence (*n =* 16), communication (*n =* 14), feedback (*n =* 8), agents within a system (*n =* 8), and non-linearity (*n =* 7). Descriptions and/or definitions of the attributes varied immensely across studies and it was difficult to know for certain if authors were referring to the same concept when using the same terminology.

Although descriptions of complexity theory varied immensely across studies, it appears authors are describing complexity theory using aspects of the theory that capture how diverse relationships and communication between agents of a system can influence unpredictable changes within the system. It comes as no surprise that descriptions often incorporate relationships, diversity, and communication. Likewise, descriptions also incorporate complexity attributes related to unpredictable changes with self-organization, emergence, and non-linearity being common in descriptions. The importance of capturing relationships and how those relationships contribute to changes in the overall system are apparent in the following examples of direct quotes of author descriptions:

- Change emerges through self-organization, defined as the mutual adjustment of behavior arising from interactions among staff as they meet immediate care demands [51].
- Complexity science suggests that organizations, such as hospitals, are complex adaptive systems. As such, a hospital is defined as a set of connected or interdependent parts or agents—including caregivers and patients— bound by a common purpose and acting on their knowledge [58].
- Complexity science, as related to healthcare, is the science of moving in a nonlinear and interactive manner where unpredictable outcomes are often realized; organizations are described as ever-changing collections of individuals and conditions in the organization; and patterns of interaction among individuals and connections are made in day-to-day practices among and between individuals [66].

Despite not knowing if authors are referring to the same thing when they use similar attributes, these three quotes of authors’ descriptions of complexity in health services research typify a common thread in the studies included in our review. In some cases, descriptions of complexity theory in health services research incorporate the theory’s ability to view communication and relationships between diverse agents in a system as supporting factors to overall changes of the system.

## Discussion

This is the first scoping review to explore how complexity theory has been incorporated into health science research. Studies incorporating complexity theory appear to be increasing in frequency. Health services researchers are primarily using complexity theory with qualitative case studies conducted in the US focused on nursing and medicine in long-term care and primary care. Quantitative and mixed methods studies using complexity theory exist, and other settings are being studied, but both to a lesser extent. Research is primarily exploratory or descriptive in nature and aimed at understanding phenomena related to interactions/relationships and management. Descriptions of complexity theory varied with 18 attributes of complexity theory across all studies in this review. The most common attributes were relationships, self-organization, and diversity. Descriptions appear to focus on aspects of complexity theory related to how diverse relationships and communication between individuals in a system may influence change.

There is notable consistency between our findings and existing reviews. Similar to Sturmberg et al.’s [20] review of complexity in family medicine general practice, we found health services researchers to be expanding how they incorporate complexity theory in research. However, this expansion has largely remained at exploratory and descriptive level of research. In a review of complexity in computer science, mathematics, and management research, Pollack et al. [19] used referencing patterns and concluded that the application of complexity theory to organizational science research using mathematical modeling techniques is uncommon. Sturmberg et al. [20] reported similar findings in family medicine general practice. Despite 14 studies in our review being quantitative, there was minimal mathematical modeling. Although some studies in our review used modeling (see for example [57, 64], mathematical modeling using complexity theory does not appear common in health services research and the use of complexity theory remains at a descriptive or exploratory level. This is not surprising since complexity theory is primarily used as an explanatory theory as opposed to predictive one [76].

Pollack et al. [19] and Sturmberg et al. [20] recommend authors move beyond metaphorical application of complexity as an observation tool. Both suggest a mathematical basis of inquiry is possible to progress complexity’s application within social sciences research. They argue a shift would enable researches to use complexity theory as a basis for quantitative modeling. Notably, neither group contends quantitative-modeling should occur without using complexity’s metaphors as building blocks for conceptual frameworks; these methodological approaches are complementary and complexity is useful for each. Although we agree with Pollack et al. [19] and Sturmberg et al. [20], we offer cautionary advice. Our findings demonstrate variation in how authors are incorporating complexity theory in health services research with a broad range of attributes being used. Thus, we align ourselves with Greenhalgh and colleagues [21] and suggest more adaptation and refinement is needed to determine how a complexity perspective can be used to answer health services research questions. That is not to say mathematical modeling is not useful. However, forgoing foundational work and shifting methodological approaches will not progress complexity’s usefulness to health services research and may only lead to more conceptual confusion. As our review suggests, there is too much variation to be certain authors are talking about, even at a metaphorical level, the same concept.

In a review of complexity in organizational science, Wallis [13] identified 20 definitions of complex adaptive systems containing 26 different conceptual components. We found authors within our review used 18 different attributes of complexity theory. Although we used different labels than Wallis, overlap exists between common attributes used in organizational science and those used in health services research. Self-organization, agents, emergence, non-linearity, and interacting/relationships were among the most common in both reviews. Likewise, descriptions that focused on how diverse relationships and communication contributed to changes within a system are predominant. Using the most common collective attributes as an indicator for what researchers consider the most applicable components of complexity theory within a social sciences context provides a foundation to begin to develop a better understanding of each concept and how it can be used to comprise a complexity theory perspective in health services research. Such foundational work is imperative. Many authors (e.g., [22, 75, 77] agree that complexity theory offers a useful perspective to answer questions of a social nature. Likewise, many authors (e.g., [53–55]) agree that descriptions of complexity theory are varied and influenced by discipline and phenomena of interest. Given complexity theory’s application in health services research is relatively new compared to other fields, health services researchers have a unique opportunity to develop the foundational conceptual perspectives that complexity theory offers health services research.

Davis and colleagues [15] suggest complexity theory is not a theory but more a perspective or way of thinking about certain phenomena. They argue that the transdisciplinary nature of a complexity perspective prevents an “off the shelf” definition and application. Although the transdisciplinary nature of complexity cannot be argued, the results of our scoping review and other reviews of complexity (i.e., [13, 20]) provide a glimpse of caution that should be considered when working with complexity. Indefinable theoretical perspectives can lead to studies with unclear or missing descriptions, implicit assumptions, and absent definitions. As a result, findings from such studies are difficult to generalize with confidence. Of course, all theories, especially transdisciplinary ones, require users to assume relationships that are, perhaps, untested. Consider Rogers’ innovation diffusion theory (a transdisciplinary theory) is the most influential theoretical perspective in the knowledge translation [78]. However, its use in knowledge translation, specifically health, requires an untested assumption that knowledge application in health is akin to classical diffusion theory [79]. Such an assumption has not limited the theory’s usefulness; however, it is worth considering in the realm of complexity how many assumptions and varied definitions are tolerable.

A lack of description of how complexity is used in original research creates challenges for drawing conclusions across health services research using review methodologies (e.g., scoping, systematic, narrative). For example, we excluded several studies where authors did not explicitly state they used complexity theory in their original manuscripts. This may have resulted in research that incorporated complexity from being excluded from our review. For example, Crabtree and colleagues have conducted a longstanding program of research using complexity theory that they outlined in a 2011 publication [80]. Such work represents a substantial contribution. However, when assessing some of Crabtree and colleagues’ original studies which form the basis of the 2011 publication (i.e. [81–85] using our inclusion/exclusion criteria, we could not include the studies because the authors did not explicitly state they used complexity theory in the original manuscripts, they did not explicitly discuss complexity theory in their original manuscripts, and it was a subsequent publication [80] that identified the studies as using complexity theory.. Notably, these studies were not captured by our search strategy because they were not indexed using medical subject headings (MeSH) related to complexity nor did they have complexity as key words or titles. Consequently, they were captured by citation searching key articles located by our database searches. While such research has the potential to advance our understanding how to use complexity to answer important health services research questions, without clear and explicit descriptions of how complexity theory was used a priori in designing a study, it is difficult to know how to use complexity theory to design future studies. Notwithstanding, papers by original authors offering a retrospective look back on their program of research from a complexity theory lens are helpful (i.e., [80, 86] but such works are difficult to integrate into reviews by other authors (e.g., this scoping review).

From this review, we stop short of recommending that complexity theory is more appropriate than other theories for incorporating into health services research. Complexity is one of many theories researchers available to health services researchers. However, the findings of our review suggest that for researchers studying factors related to relationships, communication, and diversity—specifically how these factors may contribute to change within a system—other authors have found that complexity offers an appropriate choice.

The appropriateness of complexity theory in studying systems stems from how it allows a researcher to conceptualize a system. Specifically, complexity conceptualizes a system as non-linear and dynamical. Complex systems can be understood by comparison to complicated systems. Briefly, in a complicated system, the parts that comprise the system combine in predictable, knowable ways to comprise the overall system. If one were to conceptualize a health system as complicated, it would be possible to reduce the system and study the individual to gain an understanding of the overall system. If one studied enough components, one would know how the system works and therefore how to manipulate the system. Such an approach has fallen short when studying health systems [87]. Instead, complexity theory offers a toolkit (i.e., attributes) for conceptualizing and studying health systems in different manner. Complexity brings to the forefront the unpredictable nature of a complex system. Specifically, according to complexity, systems are still comprised of agents, but those agents interact with each other. The interactions of the agents are decentralized. From these interactions, changes occur within the system that may bring about additional change. One cannot trace the original cause of the change. So, while other theories offer tools for studying systems, many are based on the assumptions that systems behave like a complicated system, are predictable, and can be understood by studying components of a system. The reason we stop short of suggesting complexity is more appropriate than other theories for studying health services research is because health systems are comprised of both complex and complicated systems. In some instances, depending on how the researcher conceptualizes the phenomena of study, theories that assume a complicated system are appropriate. However, instances where complex systems are involved, such as understanding how change may influence organizational culture, complexity theory offers an appropriate perspective.

Complexity theory is similar to other theories useful in health services research—especially theories aimed at exploring relationships in systems. Two such theories are systems theory and social network theory. Authors identify systems theory as being closely related to complexity theory [88–90]. Similar to complexity, systems theory also seeks to understand how relationships between agents of a system influence change. However, according to Phelan [89], systems theory is focused on identifying and optimizing relationship characteristics whereas complexity is focused on understanding what influences interactions so that conditions may be created to support further interactions. In essence, complexity is more exploratory whereas systems theory is more confirmatory [89]. Social network theory offers a perspective of how relationships between individuals can influence the spread of something (e.g., information, disease, innovation) within networks [91, 92]. Using social network theory, researchers can map detailed relationships between entities for the purposes of describing and predicting how network structure may influence an outcome. In essence, the focus in social network theory is the connection of agents within a system. While complexity theory also offers a perspective on connections between agents, the focus of complexity takes a less reductionist view on interactions than social network theory. Complexity theory “counsels that analytical and predictive power can only be gained by standing back—not analyzing a system in more detail” [93]. Clearly systems theory, social network theory, and many other theories are appropriate for health services research. A choice of theory depends on multiple perspectives. As such, we stop short of suggesting complexity theory is more appropriate than other theories align ourselves with Davis and Sumara [90] to suggest complexity does not rise over other theories but instead rises among them.

Variation across studies on how complexity is incorporated is expected. It is a product of intellectual grappling, experimentation, and exploration on how a complexity perspective can be incorporated to answer health services research questions. In a sense, the findings of this scoping review represent evidence that the foundational work that so many authors urge is occurring. Although we are unable to determine what is appropriate use of complexity theory in health services research, the appropriateness of variation in the early stages of complexity applied to health services research is an expected finding of this scoping review.

## Limitations

There are several limitations in our review. First, related to our search strategy, we acknowledge that not all authors will agree our search terms are integral with elements of complexity theory. We felt it necessary to take an approach of broadness during study identification, keeping with Arksey and O’Malley’s [25] framework for scoping reviews. Second, this scoping review was conducted as part of a doctoral dissertation. As such, it was conducted primarily independently (with a second reviewer when needed) and, therefore, did not benefit from a team approach to scoping methodology (see for example [26, 94]). A solitary approach has been used in scoping reviews by other doctoral candidates (e.g., [95]), however; the results would be strengthened by a team of reviewers. Third, we included only studies published in English. The effect of inclusion and exclusion in systematic reviews by language is inconclusive [96], yet there is a possibility of excluding important studies from our scoping review—most likely related to the country of research origin.

## Conclusion

Researchers are incorporating complexity theory in health services research. Researchers using complexity theory in health services research are primarily using the theory for various aspects of qualitative case studies (e.g., conceptual framework for study design, framework for data analysis) involving nursing and medicine in long-term care and primary care. Research is at the exploratory or descriptive level and focused on interactions/relationships and management. Authors have employed many attributes of complexity and descriptions often incorporate aspects of complexity theory related to how diverse relationships and communication between individuals in a system can influence change.

The overarching theme from this scoping review is variation. Although variation may be thought of as a drawback, variation may also be a product of applying a novel and malleable theory in a new context. We do not yet know how best to incorporate complexity to study phenomena in health services research and the debate is far reaching. Perhaps there is no one method to apply this theory and its malleability permits broad application? That said, authors are attempting to study important phenomena using complexity theory and are grappling with how to use this theory. Although complexity theory shows promise in health services research and health services delivery, conceptual confusion and inconsistent application hinders the operationalization of this potentially important perspective. Complexity appears particularly applicable for studying relationships and interactions between health professionals and management. However, generalizability from studies that use complexity theory, at present, is difficult due to heterogeneity and variation in reporting. Future research should include clear definitions and descriptions of complexity and how it was used in studies. In summary, more research, debate, and exploration are still needed to continue to understand how complexity theory can be incorporated in health services research.

## Abbreviations

CINAHL: cumulative index to nursing and allied health literature
EMBASE: excerpta medica database
MLIS: master of library information science

## Footnotes

1 The term *use* in this instance refers to how authors used complexity theory specifically in their study. This differs from the term *incorporated*, which we use to refer to the broader use of complexity in health services research and to encompass all of our questions.

2 The percentages in this section do not add up to 100 % because some authors used complexity theory in multiple ways.
